# Exosomes from miRNA-378-modified adipose-derived stem cells prevent glucocorticoid-induced osteonecrosis of the femoral head by enhancing angiogenesis and osteogenesis via targeting miR-378 negatively regulated suppressor of fused (Sufu)

**DOI:** 10.1186/s13287-021-02390-x

**Published:** 2021-06-07

**Authors:** Kai Nan, Yuankai Zhang, Xin Zhang, Dong Li, Yan Zhao, Zhaopu Jing, Kang Liu, Donglong Shang, Zilong Geng, Lihong Fan

**Affiliations:** 1 Department of Orthopaedics, The Second Affiliated Hospital of Xi’an Jiaotong University, No. 157 Xiwu Road, Xi’an, 710004 Shaanxi Province People’s Republic of China; 2 Department of Hepatobiliary Surgery, The First Affiliated Hospital of Xi’an Jiaotong University, Xi’an, 710004 Shaanxi Province People’s Republic of China

**Keywords:** miR-378, Exosomes, ASCs, ONFH, Sufu, Shh signaling

## Abstract

**Background:**

Local ischemia and defective osteogenesis are implicated in the progression of glucocorticoid (GC)-induced osteonecrosis of the femoral head (ONFH). Recent studies have revealed that exosomes released from adipose-derived stem cells (ASCs) play important roles in ONFH therapy. The present study aimed to investigate whether exosomes derived from miR-378-overexpressing ASCs (miR-378-ASCs-Exos) could promote angiogenesis and osteogenesis in GC-induced ONFH.

**Methods:**

In vitro, we investigated the osteogenic potential of miR-378-ASCs-Exos on bone marrow stromal cells (BMSCs) by alkaline phosphatase staining and western blotting. The angiogenic effects of miR-378-ASCs-Exos on human umbilical vein endothelial cells (HUVECs) were examined by evaluating their proliferation, migration, and tube-forming analyses. We identified the underlying mechanisms of miR-378 in osteogenic and angiogenic regulation. In addition, an ONFH rat model was established to explore the effects of miR-378-ASCs-Exos through histological and immunohistochemical staining and micro-CT in vivo.

**Results:**

Administration of miR-378-ASCs-Exos improved the osteogenic and angiogenic potentials of BMSCs and HUVECs. miR-378 negatively regulated the suppressor of fused (Sufu) and activated Sonic Hedgehog (Shh) signaling pathway, and recombinant Sufu protein reduced the effects triggered by miR-378-ASCs-Exos. In vivo experiments indicated that miR-378-ASCs-Exos markedly accelerated bone regeneration and angiogenesis, which inhibited the progression of ONFH.

**Conclusion:**

Our study indicated that miR-378-ASCs-Exos enhances osteogenesis and angiogenesis by targeting Sufu to upregulate the Shh signaling pathway, thereby attenuating GC-induced ONFH development.

## Introduction

Osteonecrosis of the femoral head (ONFH) is a progressive and refractory orthopedic disease [1]. The overuse of glucocorticoids (GC) is the most common cause of nontraumatic ONFH [2], which occurs at younger ages, and most symptomatic patients require surgery [3]. Current therapies for pre-collapsed ONFH are usually unsatisfactory owing to incomplete prevention of compromised subchondral microcirculation, endothelial dysfunction, and deficient bone repair [4]. Thus, effective measures are needed to enhance angiogenesis and osteogenesis in the early stages of GC-induced ONFH.

Cell transplantation technologies offer a promising approach for bone regeneration. Bone marrow stromal cells (BMSCs) are the most widely used stem cells for the treatment of ONFH [5]. In a meta-analysis study, the addition of BMSCs to core decompression achieved improved clinical outcomes and lower rates of disease progression in patients with ONFH [6]. Many studies have demonstrated that the application of BMSCs accelerates the repair process by stimulating angiogenesis and osteogenesis [7]. However, BMSCs have limitations because of insufficient cell numbers and considerable donor site morbidity [8]. Adipose tissue-derived stem cells (ASCs) have attracted attention owing to their body-wide storage, easy acquisition, and expansion. Recent studies have also identified that ASCs possess the ability to undergo osteogenic and angiogenic differentiation, suggesting an advantage for their application in bone regeneration [9]. Nevertheless, drawbacks to direct stem cell transplantation include low survival rates, genetic modification, and tumor formation [10].

Recently, accumulating evidence has indicated that the therapeutic effect of stem cell transplantation is mediated through exosome secretion [11]. Exosomes are extracellular lipid-structure vesicles with a diameter of 50–100 nm, formed by stem cells. These vesicles transmit bioactive proteins, lipids, and RNAs to target cells for intercellular communication, featuring low tumorigenicity and immunogenicity [12]. Exosomes have been reported to exert positive or negative effects on bone diseases during physiological and pathological processes. Previous clinical studies have shown that exosomes reduce blood urea nitrogen and creatinine, as well as improve estimated glomerular filtration rates [13]. Exosomes secreted by BMSCs can prevent early GC-induced ONFH by enhancing osteogenesis and angiogenesis [14, 15]. The variable proportions of the most representative miRNAs in ASCs- and BMSCs-derived exosomes suggest that exosomes might deliver different information into their microenvironment [16]. ASC-derived exosomes (ASCs-Exos) have been shown to effectively promote cell migration, angiogenesis, and neovascularization in ischemic diseases [17]. However, the effects and mechanisms of ASCs-Exos in GC-induced ONFH remain unknown. The lack of osteoinductive effects of ASCs can be a limiting factor in the treatment of ONFH [18]. To better apply ASCs-Exos for clinical applications, the simultaneous potential of angiogenesis and osteogenesis needs further enhancement.

MicroRNAs (miRNAs) are small noncoding RNAs that are important for bone remodeling such as angiogenesis, osteoclastogenesis, and osteogenesis and are involved in the pathogenesis and treatment of ONFH [19]. The transfer of specific miRNAs enhances the osteogenesis and angiogenesis ability of exosomes because exosomal miRNAs have been demonstrated to regulate recipient cell functions [20]. miR-378 has been reported to be closely associated with cell proliferation, angiogenesis, and metastasis. MSCs transfected with miR-378 showed higher viability and angiogenesis under hypoxic-ischemic conditions [21]. Additionally, miR-378 can directly enhance BMP2-induced osteogenic differentiation of C2C12 cells [22]. Taken together, the enrichment of miR-378 in ASCs-Exos might enhance the therapeutic efficiency of ASCs-Exos in GC-induced ONFH. In the present study, we investigated the effects of ASCs-Exos on angiogenesis and osteogenesis in GC-induced ONFH and explored the underlying mechanisms.

## Materials and methods

### Cell culture

ASCs utilized in experiments reported here were obtained from normal 8–10 weeks rats and expanded in culture as previously described [23]. Briefly, minced epididymal fat pads were digested with 2 mg/mL collagenase type 1 (Worthington, Lakewood, NJ, USA) prepared in phosphate-buffered saline containing 2% penicillin/streptomycin (P/S). Once isolated, ASCs were plated at a density of 1 × 10^5^ cells/well in a 6-well plate and incubated in Dulbecco’s modified Eagle’s medium (DMEM) supplemented with 10% fetal bovine serum (FBS) containing 100 U/mL P/S at 37 °C, under 5% CO_2_. The medium was changed every second day until the confluence of ASCs reached 80–90%. The third to fifth passages of ASCs were used for experiments.

BMSCs were derived from rat bone marrow as previously described [24]. Briefly, BMSCs were isolated using the Ficoll density gradient centrifugation method, and cultured in Essential Medium Alpha Medium (α-MEM) containing 2 mM L-glutamine, 10% FBS, and 100 U/mL P/S at 37 °C under 5% humidified CO_2_. After 24 h, non-adherent cells were removed, and adherent cells were further cultured until approximately 80% of the cells were fused in the complete medium.

Human umbilical vascular endothelial cells (HUVECs) were purchased from the China Center for Type Culture Collection and cultured in Medium 199 supplemented with antibiotics (50 U/mL P/S), 50 mL endothelial cell growth supplement, and 10% FBS. Cells were incubated at 37 °C in a humidified atmosphere containing 5% CO_2_.

### Cell transfection

The miR-378 mimics (GenePharm, China) were transfected into ASCs using Lipofectamine 2000 (Invitrogen, USA) according to the manufacturer’s protocol. First, ASCs were cultured to 70–90% confluence in a 6-well plate at a density of 1 × 10^5^ cells/well. Then, 3 μL of miR-378 mimic was diluted in 300 μL Opti-MEM and Lipofectamine 2000 in a 1:1 ratio. Finally, the miRNA-lipid complex was added to ASCs and incubated for 48 h at 37 °C.

### Isolation and identification of exosomes

Exosomes were purified from ASCs and miR-378-ASCs by differential ultracentrifugation as previously described [25]. First, the supernatant of ASC culture medium was centrifuged at 300*g* for 20 min to remove cells and cell fragments were removed at 2000*g* for 20 min. After filtration, ASCs-conditioned medium was obtained. Then, this was centrifuged at 1000*g* for 30 min to harvest a concentrated liquor of ASCs-exosomes. After centrifugation at 10^5^*g* for 120 min, the lower liquid layer was diluted with PBS and centrifuged at 1000*g* for 30 min. After washing three times in PBS, the exosomes were collected and frozen at − 80 °C for experiments. All centrifugation steps were performed at 4 °C.

Transmission electron microscopy (TEM) was used to examine the morphology of ASCs-exosomes and miR378-ASCs-exosomes. In brief, the purified exosomes suspension was diluted to 500 μg/L and fixed with glutaraldehyde. Then 20 μL of fixed solution was added to the copper network and stained with 3% phosphotungstic acid solution for 5 min. After drying, exosomes ultrastructure was observed by TEM (Hitachi, Japan).

The exosomes were then diluted with PBS. The suspension was examined using Nanosight NS300 (Malvern Panalytical, Malvern, UK) to analyze particle sizes and concentrations of ASCs-exosomes or miR378-ASCs-exosomes.

### CCK8 assay for BMSCs proliferation

The CCK8 kit (Dojindo, Kumamoto, Japan) was used to assay BMSCs proliferation. According to the manufacturer's instruction and other literature [26], BMSCs suspensions (5000 cells/well) were inoculated into 96-well plates and cultured with saline, 10 μM dexamethasone (DEX), DEX+ASCs-Exos, or DEX+miR378-ASCs-exos (50 μg/mL). The concentration of exosomes was set according to previous papers on osteogenic and angiogenic promotion in MSCs [26, 27]. Each group contained five wells for data verification. Then, 10 μL CCK8 reagent was added to each well and cultured at 37.5 °C in a 5% CO_2_ incubator. OD values were measured at 450 nm on days 0, 1, 2, 3, 4, and 5. The average optical density of five wells was defined as a data point.

### Tube formation assay

To evaluate the capacity of miR378-ASCs-exosomes in terms of angiogenesis, tube formation assays were performed according to a previously reported protocol [28]. First, HUVECs were cultured with different serum-free medium (10 μM DEX, DEX+ASCs-Exos, and DEX+miR378-ASCs-Exos (50 μg/mL)) in a 6-well plate for 24 h. At the same time, Matrigel (Corning, USA) was moved from − 20 °C to 4 °C and placed on ice overnight for liquefaction in advance. Then a 48-well plate was coated with 100 μL Matrigel on ice. For polymerization, the plate was incubated for one hour at 37 °C. The preconditioned HUVECs were seeded onto Matrigel at 1 × 10^5^ cells/well after washing with PBS. After incubation for 12 h, capillary-like structures and the number of capillary-like rings were observed under an optical microscope (Olympus, Japan). Subsequently, photomicrographs were taken from randomly selected five fields in each group using the same microscope. The total tube length in each group was analyzed using ImageJ software.

### Transwell migration assay

To detect the effect of miR-378-ASCs-Exos on the migration capacity of HUVECs, Transwell migration assays were performed as described previously [29]. In brief, preconditioned HUVECs (saline, 10 μM DEX, DEX+ASCs-Exos, and DEX+miR378-ASCs-Exos (50 μg/mL)) were plated on the filter membrane of the Transwell insert. As a chemoattractant, complete culture medium was added to the bottom of the lower chamber. After 48 h, the membrane was fixed with 70% ethanol and stained with 2% crystal violet (Abcam, UK). Finally, the number of attached cells was counted from five randomly selected fields in each group under an optical microscope. Averages of cell counts were obtained and compared with that of the control group to calculate relative cell numbers.

### Alkaline phosphatase (ALP) staining

Third-generation BMSCs were cultured at a density of 1 × 10^11^ cells/L in a 24-well plate. Then saline, 10 μM DEX, DEX+ASCs-Exos, or DEX+miR378-ASCs-Exos (50 μg/mL) were added to the BMSCs medium. On the 7th day of culture, the cells were fully lysed with 0.2% Triton X-100 (Sigma-Aldrich, USA). After washing twice with PBS and centrifuging at 12,000 rpm for 10 min, the supernatant was collected to evaluate ALP activity using a BCIP/NBT alkaline phosphatase color development kit (Solarbio, Beijing, China) at a wavelength of 405 nm.

### Quantitative real-time polymerase chain reaction (RT-qPCR) for miR-378 expression

Total RNA of miR378-ASCs-Exos and ASCs-Exos was extracted using the TRIzol method (Invitrogen, USA), and RNA purity was measured using a spectrophotometer. The miR-378 was reverse-transcribed to cDNA using the Reverse Transcription Kit PLUS (EZ Bioscience) and detected using the SYBR Green fluorescent real-time PCR kit (Invitrogen, USA). The primers (BioTNT, Shanghai, China) were as follows: miR-378, sense 5′-CTGAGACTGGACTTGGAGTC-3′, antisense 5′-GTGCAGGGTCCGAGGT-3′. The miRNA expression was calculated using the 2^-ΔΔCt^ method.

### Western blotting and antibody

To further detect the expression of CD63, CD81, BMP-2, RUNX2, ANG-1, VEGF, Sufu, Ptch1, and Gli1, western blotting was performed according to the manufacturer’s protocol [30]. Briefly, cell RIPA lysis buffer (Beyotime, China) was used to extract total protein. A BCA protein assay kit (Beyotime, China) was used to detect protein concentrations according to the manufacturer’s specifications. Equal amounts of protein extracts were separated by SDS–PAGE and transferred electrophoretically to PVDF membranes (Millipore, Bedford, USA), which were blocked with 5% non-fat dry milk for 2 h. Then, the membranes were incubated for 12 h at 4 °C with primary antibody (Abcam, USA). The membrane was then re-probed with appropriate secondary antibodies conjugated to horseradish peroxidase for 1 h. Western chemiluminescent ECL reagent (Tiangen, China) was used to detect secondary antibodies. Finally, the results were recorded using ImageJ software.

### Dual-luciferase reporter gene assay

TargetScan (http://www.targetscan.org/vert_71/) was used to analyze relationships involving Sufu and miR-378. Luciferase reporter assays were performed to verify such relationships. Sufu dual-luciferase reporter gene vector and mutated vector involving the miR-378 binding site, namely, PGLO-Sufu wild type (WT) and PGLO-Sufu mutant type (MUT), were subsequently constructed. The two recombinant vectors were co-transfected into HEK-293T cells with the miR-378 mimic or miR-NC. The Dual-Luciferase Reporter Assay System (Promega, USA) was used to assay the luciferase activity.

### Establishment of animal ONFH model and treatment protocol

Mature male SD rats (age: 8–10 weeks; body weight: 180–340 g) were utilized for this experiment, all were obtained from the Experimental Animal Center of Xi’an Jiaotong University, China. Before experiments, they were subjected to standard laboratory diets and housed under standard conditions. GC-induced ONFH in rats was developed according to previously published protocols [31]. The Animal Ethics Committee of Xi’an Jiaotong University approved all experimental protocols. Procedures were conducted in accordance with the NIH Guide for the Care and Use of Laboratory Animals. Briefly, 80 rats were divided into four groups (n = 20) by using the random number table method. An equivalent dose of saline was administered injected to the control group. The other groups received two doses via tail intravenous injections, each of 20 μg/kg body weight of lipopolysaccharide (LPS, Sigma, USA), at 24 h intervals. Subsequently, the rats received another three doses of intramuscular injections of methylprednisolone (MPS, 40 mg/kg) at intervals of 24 h each. After each MPS injection, the ASCs-exos and miR-378-ASCs-exos groups were subjected to administration of 100 μg exosomes (resuspended in 200 μL PBS) via intravenous tail injection. The model and control groups were injected with equal volumes of PBS.

### Micro-computed tomography (μ-CT) imaging

After four weeks of feeding, all rats were sacrificed and femur heads were extracted and fixed in 4% paraformaldehyde solution (pH 7.4) overnight. The femoral metaphysis was scanned by the micro-CT (Y.Cheetah, Germany). The scan resolution was set at 10 μm, and three-dimensional images were rebuilt to display the structure of trabecular bone. The most relevant parameters were analyzed, including trabecular thickness (Tb.Th), trabecular separation (Tb.Sp), bone volume per tissue volume (BV/TV), and trabecular number (Tb.N).

### Immunohistochemistry and histopathological staining

After decalcification and fixation in paraffin, the femur heads were cut into 5-μm-thick sections. The slices were then stained with hematoxylin and eosin (H&E) to evaluate the trabecular structure and osteocyte lacunae. In addition, VEGF, CD31, and RUNX2 immunohistochemical staining were used to evaluate angiogenesis and osteogenesis, respectively. To quantify the intensities of immunostaining in each group, images were analyzed using Image-Pro Plus v.6.0.

### Statistical analysis

All data are presented as means ± SD. One-way analysis of variance (ANOVA) was performed to test differences in means in multiple groups. Dunnett’s test was used to compare mean values between two groups. Statistical analysis was performed using SPSS v.20.0. Statistical significance was set at P < 0.05.

## Results

### Characterization of exosomes

**Fig. 1:**
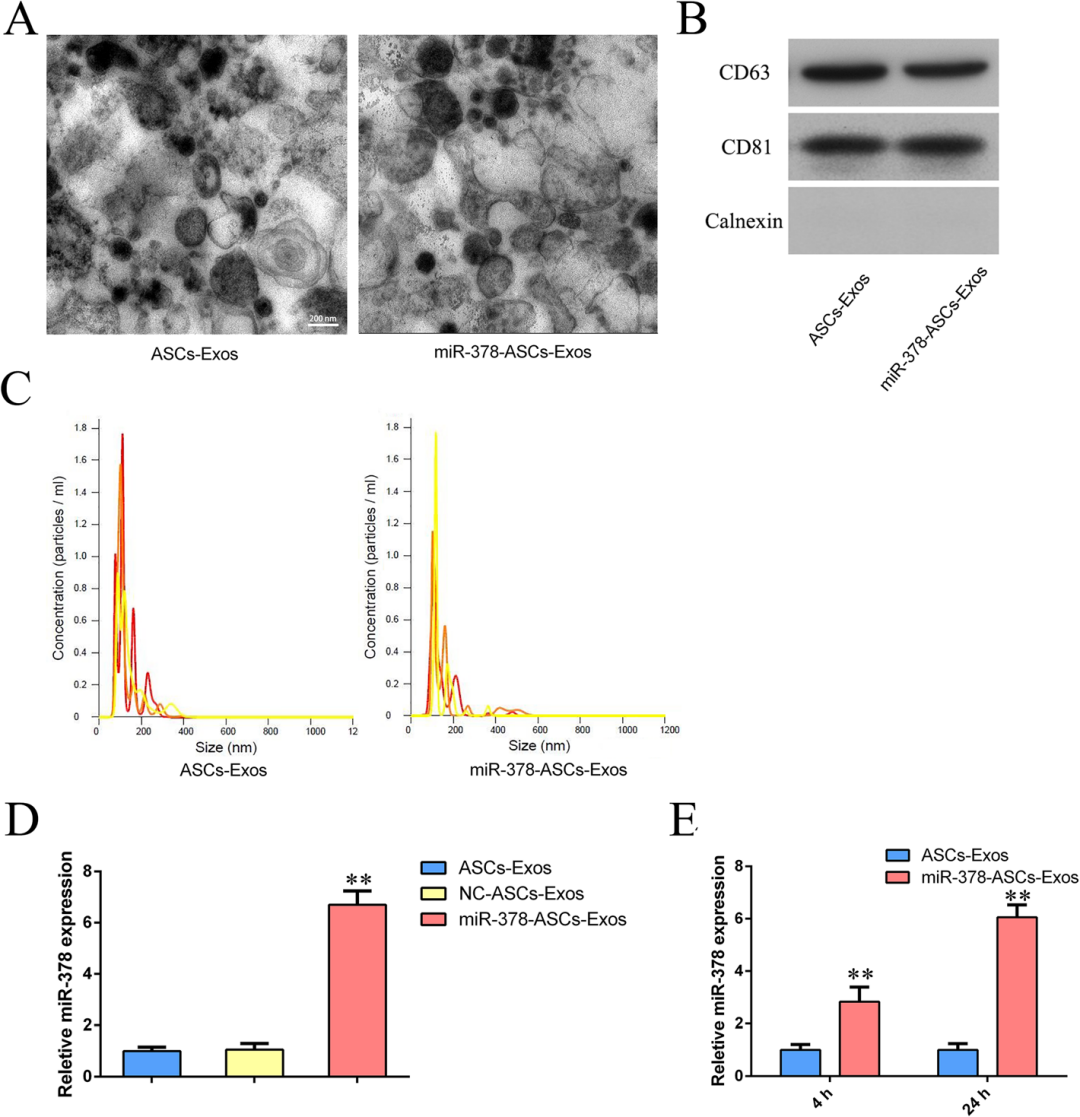
Characterization of exosomes and expression of miR-378 in ASCs-Exos. **a** TEM images show the ultrastructure of ASCs-derived exosomes. **b** Expression of the exosome markers CD63, CD81, and Calnexin confirmed by western blotting. **c** The size distribution of ASCs-Exos and miR-378-ASCs-Exos was determined by nanoparticle tracking analysis (NTA). **d** Levels of miR-378 in exosomes secreted from ASCs with or without miR-378 overexpression. **e** The levels of miR-378 in BMSCs were measured by RT-qPCR after miR-378-ASCs-Exos treatment. **P < 0.01 compared with the ASCs-Exos group

To objectively characterize exosomes from miRNA-378-modified ASCs and ASCs, TEM, western blotting, and nanoparticle tracking analysis (NTA) were performed. TEM demonstrated that the morphology of exosomes was rounded and their diameters were approximately 50–170 nm (Fig. 1a), which was in accord with the results of NTA (Fig. 1c). Western blotting showed that characteristic cell-surface markers, including CD63 and CD81, were positive in miR-378-ASCs-Exos, whereas calnexin was negative, which was similar to that of ASCs-Exos (Fig. 1b). However, as shown in Fig. 1d, the expression level of miRNA-378 measured by RT-qPCR in miR-378-ASCs-Exos was significantly higher than that of isolated ASCs-Exos (P < 0.01). RT-qPCR analysis also revealed that the expression of miR-378 in BMSCs was remarkably elevated after miR-378-ASCs-Exos treatment in a time-dependent manner (P < 0.01) (Fig. 1e).

### miR-378-ASCs-Exos increased cell migration, proliferation, and angiogenic capacity

**Fig. 2:**
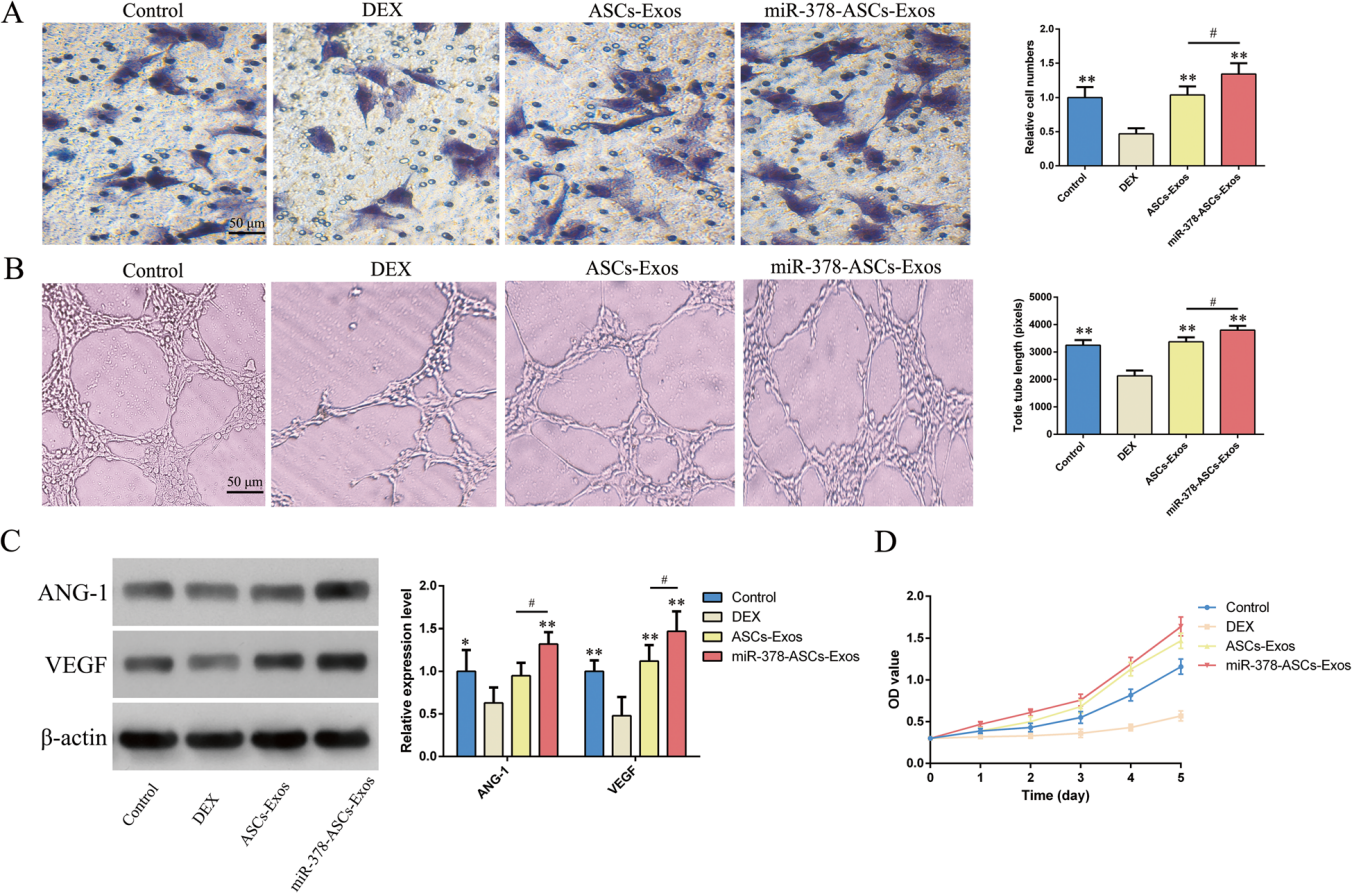
Effects of miR-378-ASCs-Exos on cell migration, proliferation, and angiogenetic capacity. **a** Cell migration ability measured by Transwell assays. **b** Tube formation capability detected in HUVECs endothelial cells stimulated with 5 μg/mL ASCs-Exos or ASCs-miR-378-Exos 24 h. **c** Western blot and quantification of ANG-1, VEGF expression in HUVECs. **d** Cell proliferation of HUVECs treated with DEX with or without miR-378-ASCs-Exos, as evaluated by CCK-8 analysis. *P < 0.05, **P < 0.01 compared with the DEX group. ^#^P < 0.05, ^##^P < 0.01

As shown in the Transwell assays, cell migration ability was significantly decreased in the DEX group compared with that in the control group (P < 0.01). In contrast, the inhibitory effect of DEX was restored by exosomes (P < 0.01), which was more significant in the mir-378-ASCs-Exos group (P < 0.05) (Fig. 2a). In addition, the results of CCK-8 assays revealed that miR-378-ASCs-Exos eliminated the DEX suppressive effect (P < 0.01) and improved the viability of HUVECs more significantly than ASCs-Exos (Fig. 2d). To further explore the capacity for angiogenesis in vitro, greater significant vascularization effects were observed microscopically by stimulation with miR-378-ASCs-Exos compared with the DEX group, which was similar to the results of quantification of formed tube lengths (P < 0.01) (Fig. 2b). Western blotting revealed that the expression of relevant angiogenic factors ANG-1 and VEGF was remarkably improved in HUVECs stimulated with miR-378-ASCs-Exos (P < 0.01) (Fig. 2c).

### miR-378-ASCs-Exos promoted osteogenic differentiation of BMSCs in vitro

**Fig. 3:**
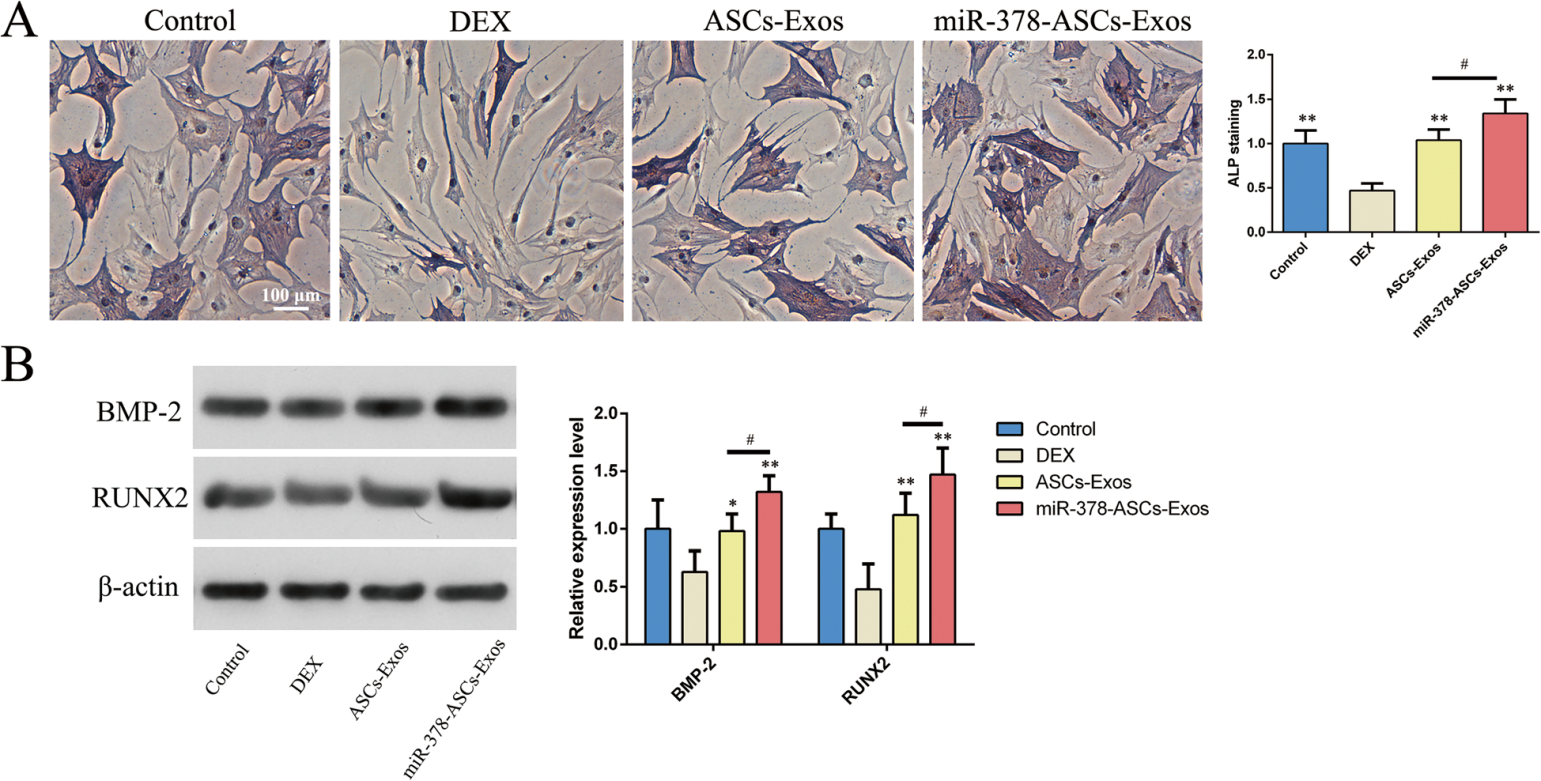
miR-378-ASCs-Exos promoted osteogenic differentiation of BMSCs in vitro. **a** miR-378-ASCs-Exos at 50 μg/mL was delivered into BMSCs. ALP staining on day 7. **b** The expression levels of osteogenesis-related factors including BMP2, RUNX2 were evaluated by western blot in the different groups. *P < 0.05, **P < 0.01 compared with the DEX group. ^#^P < 0.05

According to the results of ALP staining and activity presented in Fig. 3a, it was apparent that the osteogenic differentiation of BMSCs was attenuated in the isolated DEX group. However, this downregulation was remarkably reversed by miR-378-ASCs-Exos stimulation (P < 0.01). In addition, the expression of osteogenesis-related factors including BMP2 and RUNX2 was also detected by western blotting. As shown in Fig. 3b showed, upregulation of protein expression was observed to be statistically significant in the exosomes group, especially with miR-378-ASCs-Exos stimulation (P < 0.01).

### miR-378 promoted angiogenesis and osteogenesis by downregulating Sufu and activating Shh activity

**Fig. 4:**
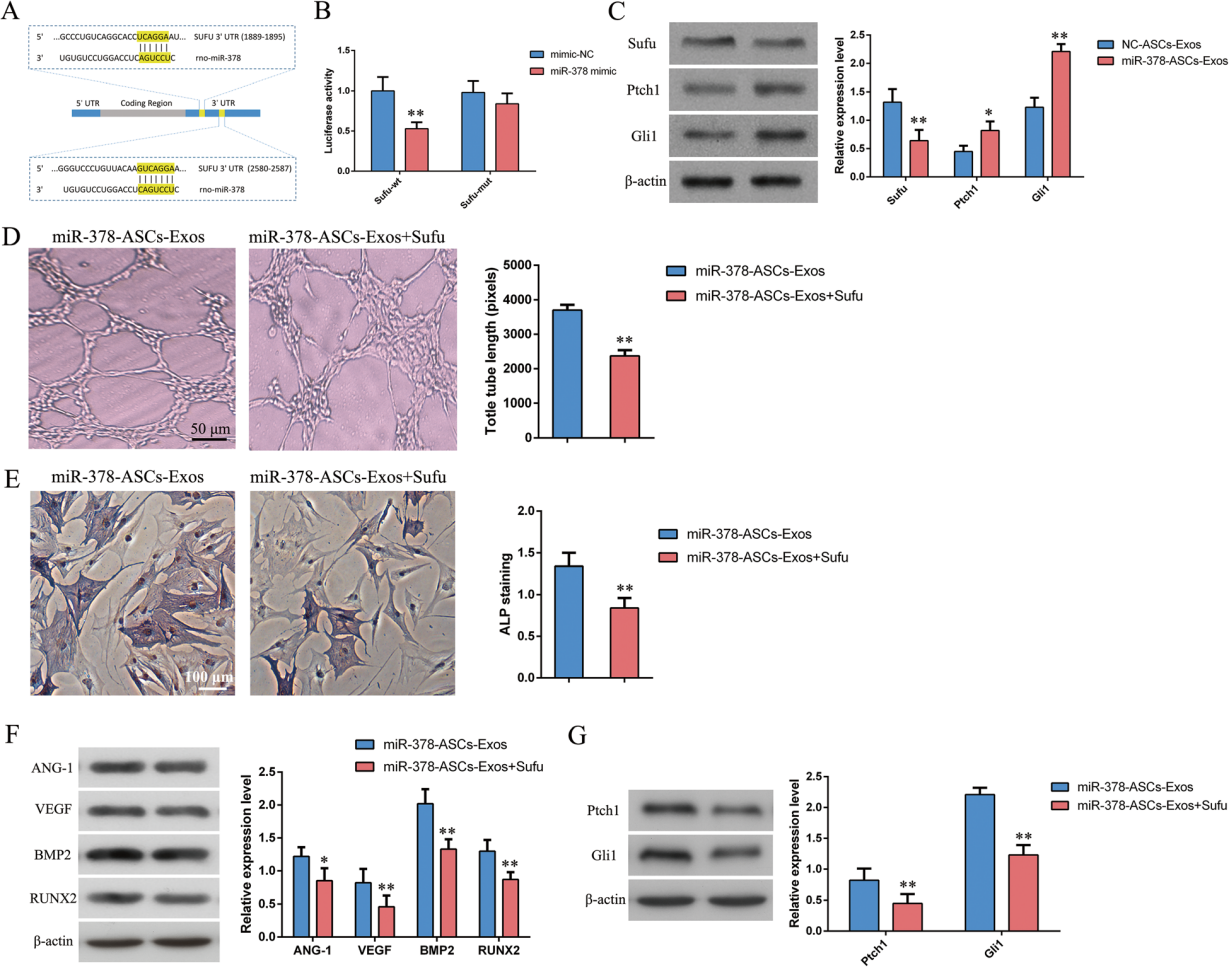
miR-378 promoted angiogenesis and osteogenesis by down-regulating Sufu and inducing Shh activity. **a** The binding sites of miR-378 and Sufu were validated using an online website. **b** The targeting relationship between miR-378 and Sufu was verified suing dual luciferase reporter gene assay. **c** Expression patterns and extent of Shh signaling pathway-related factors (Sufu, Ptch1, and Gli1) identified by Western blot analysis. **d** Tube formation assay for detecting tube-forming ability of HUVECs in the different groups. **e** Expression of ALP was determined by ALP staining in different groups. **f** Expression patterns of vascular endothelial growth factors (ANG-1, VEGF) and osteogenesis-related factors (BMP2, RUNX2) were measured by Western blot analysis. **g** Western blotting of Ptch1 and Gli1 expression. *P < 0.05, **P < 0.01

Based on the microRNA.org website, the specific binding sites of miR-378 were found in the 3′UTR of Sufu (Fig. 4a). Luciferase reporter assays revealed that miR-378 repressed luciferase activity in the WT 3′UTR of Sufu (P < 0.01), but not in the MUT 3′UTR (Fig. 4b). The expression of Sufu and Shh signaling pathway-related factors was detected by western blot analysis (Fig. 4c). These results revealed that treatment with miR-378-ASCs-Exos negatively regulated the expression of Sufu, while promoting the expression of Shh signaling pathway-related factors (Ptch1 and Gli1) (P < 0.01). Osteogenetic effects were observed with ALP staining, and the results indicated that recombinant Sufu protein suppressed osteogenesis caused by miR-378-ASCs-Exos (P < 0.01) (Fig. 4e). According to the results of tube formation assays, the angiogenic ability of HUVECs in the miR-378-ASCs-Exos group was significantly downregulated by additional Sufu stimulation (P < 0.01) (Fig. 4d). To further detect the expression of relevant factors related to angiogenesis (VEGF and ANG-1) and osteogenesis-related factors (BMP2 and RUNX2), western blot analysis was performed. Figure 4f demonstrates that Sufu treatment repressed the expression of relevant proteins involved in angiogenesis and osteogenesis in the miR-378-ASCs-Exos group. Further analysis demonstrated that the increased expression of Shh signaling pathway-related factors induced by miR-378-ASCs-Exos was significantly reversed by Sufu (P < 0.01) (Fig. 4g). These findings indicated that miR-378 promotes angiogenesis and osteogenesis by downregulating Sufu and activating Shh pathways.

### miR-378-ASCs-Exos enhanced neovascularization in a rat model of ONFH

**Fig. 5:**
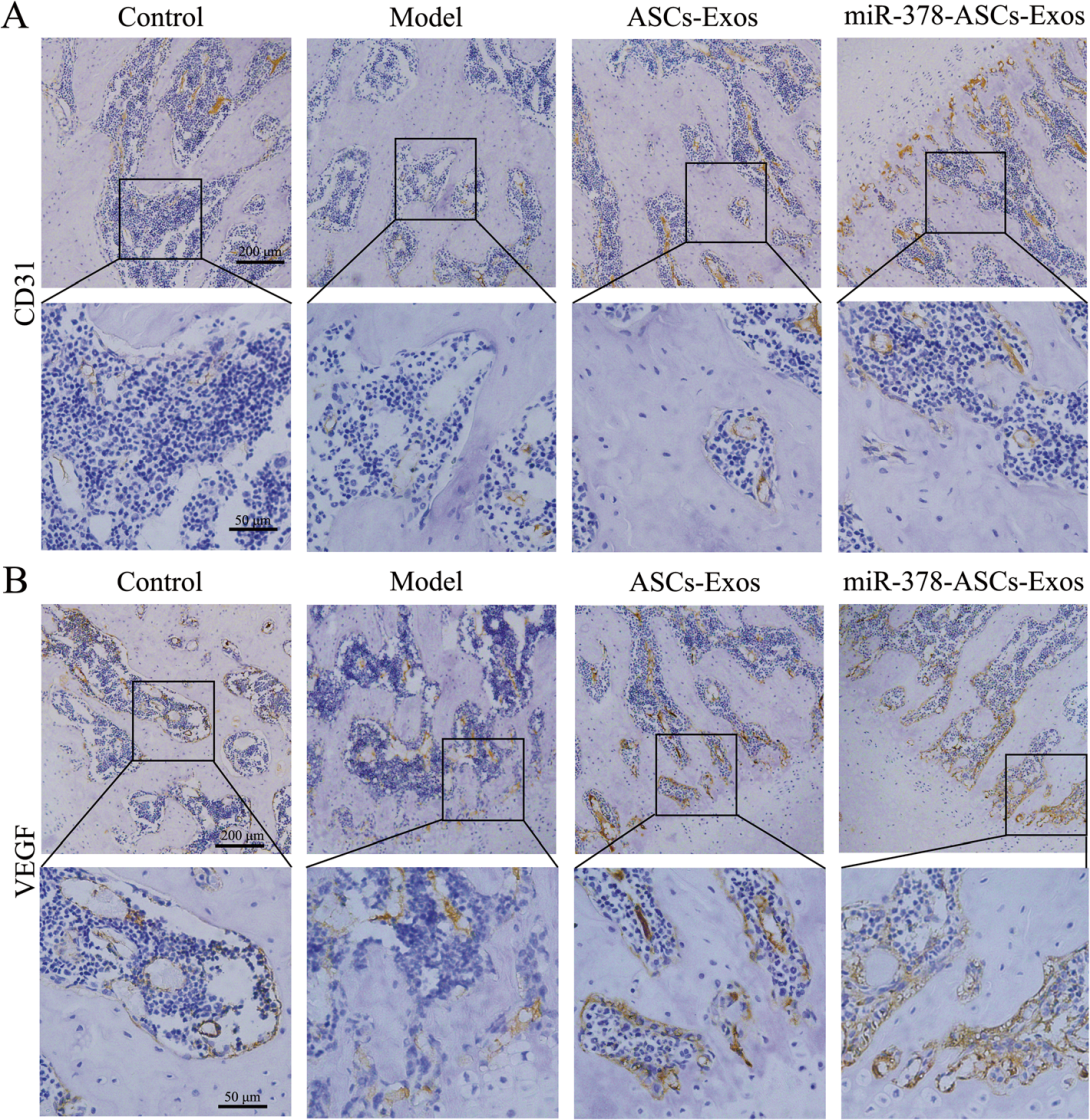
Assessment of angiogenesis in the femoral head. **a** Immunohistochemical analysis of CD31. **b** Immunohistochemical analysis of VEGF

The angiogenetic effects of miR378-ASCs-Exos in the femoral head were evaluated by immunohistochemical staining for CD31 and VEGF. Immunohistochemical staining revealed that many single or clusters of endothelial cells expressing CD31 were observed in microvessels in the exosomes group (Fig. 5a). Expression in the miR378-ASCs-Exos group was more significant. Compared with the control group, the expression of VEGF was remarkably reduced in the model group. In contrast, the administration of ASCs-Exos with or without miR-378 significantly reversed the decreased expression of VEGF (Fig. 5b). These results demonstrated that miR-378-ASCs-Exos can promote neovascularization in a rat model of ONFH.

### miR-378-ASCs-Exos promoted osteogenesis in vivo

**Fig. 6:**
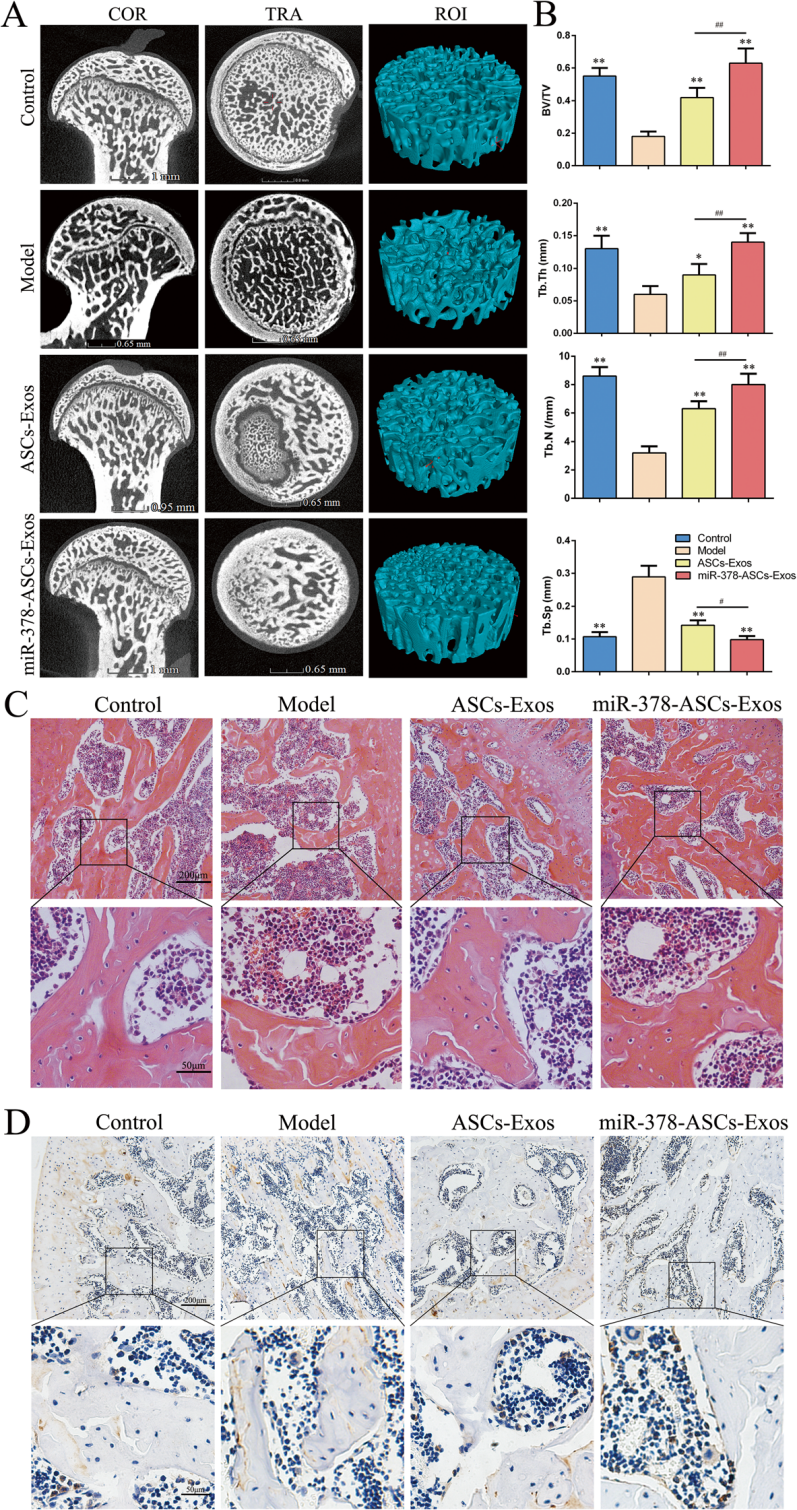
The osteogenesis-promoting effects of miR-378-ASCs-Exos in the rat model of GC-induced ONFH. **a** Reconstructed coronal and transverse images of bones. **b** Quantitative analyses of trabecular thickness (Tb.Th), trabecular separation (Tb.Sp), bone volume per tissue volume (BV/TV), and trabecular number (Tb.N) in the different treatment groups. **c** H&E staining of femoral heads in rats receiving different treatments. **d** Immunohistochemical staining for RUNX2 in samples from the different treatment groups. *P < 0.05, **P < 0.01 compared with the model group. ^#^P < 0.05

To detect the osteogenesis-promoting effects of miR-378-ASCs-Exos in vivo, three-dimensional micro-computed tomography (3D-μCT), immunohistochemical staining for RUNX2, and H&E staining were performed to evaluate the bone tissue in a rat model of GC-induced ONFH. μCT images revealed that significant trabecular damage to the femoral head in the model and ASCs-Exos groups (Fig. 6a). However, with the administration of miR-378-ASCs-Exos, this damaging effect was significantly reversed. Furthermore, quantitative analyses also demonstrated that the microstructural parameters (Tb.Th, BV/TV, Tb.N, and Tb.Sp) of the miR-378-ASCs-Exos group were remarkably superior to those of the model group (P < 0.01), with almost no difference to the control group (Fig. 6b). The results of H&E staining showed that the trabecular bone of the model group was exiguous, and more extensive cystic degeneration was observed in the subchondral area. Conversely, exosome treatment effectively suppressed osteonecrosis (Fig. 6c). It is noteworthy that the osteogenetic effect was elevated more significantly in the miR-378-ASCs-Exos group than in the ASCs-Exos cohort. Furthermore, among the four groups, the expression of RUNX2 was highest after miR-378-ASCs-Exos treatment according to immunohistochemical staining, indicating a superior osteogenesis-promoting effect (Fig. 6d). The results showed that miR-378-ASCs-Exos enhance bone formation in a rat model of ONFH.

## Discussion

Stem cell therapies have emerged as promising strategies for the treatment of GC-induced ONFH because their ability to promote angiogenesis, alleviate oxidative stress, reduce adipogenesis and enhance osteogenesis [32]. Angiogenesis is essential for osteogenesis during skeletal development, remodeling, and regeneration [33]. Osteoblast precursors produce pro-angiogenic factors and are directly associated with invading blood vessels [34]. Blood vessels not only supply the skeletal system with oxygen, nutrients, specific hormones, and growth factors, they also play an essential role in the recruitment of osteoblast precursor cells [35]. Recent studies demonstrated that type-H vessels located near the growth plate in the metaphysis could mediate the growth of bone vasculature, maintain osteoprogenitors, and couple angiogenesis to osteogenesis [36]. ASCs may provide superior cellular therapeutics potential involving BMSCs for treating ONFH [37]. BMP2-/VEGF-transfected ASCs show enhanced new bone formation and angiogenesis in large bone defects [38]. ASCs-Exos have the potential to improve graft retention by promoting angiogenesis [39]. Meanwhile, ASCs-Exos can promote BMSCs proliferation, migration, and osteogenesis, indicating their therapeutic capacity for bone regeneration [40]. In the present study, we found that miR-378-ASCs-Exos significantly inhibited the progression of GC-induced ONFH.

miR-378 has been indicated to be an important regulator of a variety of cellular and organic metabolic processes. Direct evidence has shown that miR-378 is associated with tumor development, angiogenesis, and metastasis [41, 42]. It has also been found that miR-378 can directly affect VEGF expression by competing for the same seed region in the VEGF 3′-UTR [43]. Moreover, miR-378 regulates the proangiogenic and paracrine capacities of CD34^+^ progenitor cells activated in acute myocardial infarction [44]. Experimental studies have shown that miR-378 can significantly promote cell survival and neovascularization in hypoxic-ischemic environments [21]. We integrated its proangiogenic function with ASCs exosomes to improve revascularization for regenerative purposes. Here we found that miR-378-ASCs-Exos could promote cell migration and tube-forming capacity otherwise impaired by GCs. In addition, miR-378-ASCs-Exos enhanced the expression of angiogenesis-related genes such as VEGF and ANG1. Vascular endothelial cells and blood vessels were also observed surrounding newly formed bone tissue in the miR-378-ASCs-Exos group following immunohistochemical staining for CD31. These results indicated that miR-378-ASCs-Exos can act as an efficient therapeutic intervention for GC-induced ONFH by improving vascularization.

Previous studies have confirmed that the addition of ASCs-Exos to mosteogenic medium strengthens the osteoinductive capacity of BMSCs [40]. Meanwhile, the differentially expressed exosomal miRNAs play an important role ASCs osteogenesis [45]. Exosomes rich in miR-130a-3p have already been shown to positively regulate osteogenic differentiation of ASCs [46]. miR-378 enhances osteogenic differentiation of BMSCs and C2C12 cells and attenuates high glucose-suppressed osteogenic differentiation of MCT3T3-E1 cells [22, 47, 48]. Therefore, transfection of miR-378 into ASCs also has the potential to enhance the osteogenic capacity of ASCs-Exos. Here we found that miR-378-ASCs-Exos strengthened the osteogenic differentiation of BMSCs under the negative impact of GC. In addition, bone remodeling was partially restored in the miR-378-ASCs-Exos group in vivo. These findings provide evidence that miR-378-ASCs-Exos can promote osteogenesis and improve GC-induced ONFH.

We further investigated the potential mechanisms underlying miR-378-ASCs-Exos in promoting angiogenesis and osteogenesis. According to previous studies, miR-378 targets the 3′-UTR of Sufu in vertebrates [49], which is recognized as a negative regulator of the Sonic Hedgehog (Shh) signaling pathway [48]. The Shh signaling is a key signaling pathway engaged in the development of many tissues and organs, such as bone and cartilage. Binding of the Shh ligand to Ptch1 relieves the inhibition of Smo, followed by activation of Gli transcription factors, which translocate to the nucleus and promote the expression of target genes [50]. Sufu exerts its functions by retaining Gli1, the nuclear effector protein of Shh signaling, to prevent it from being translocated into the nucleus to trigger the transcriptional program [51]. In this study, we demonstrated that miR-378 was successfully transferred into recipient cells by exosomes, thereby downregulating Sufu and activating the Shh signaling pathway.

Studies in mice have shown that the Shh pathway regulates osteoblast differentiation and proliferation of mesenchymal progenitor cells by elevating Runx2 expression [52]. It has also been confirmed that the activation of Shh signaling causes increased matrix deposition in normal fracture repair [53, 54]. Meanwhile, Shh pathway stimulation promotes neovascularization by secreting angiogenic growth factors, which contribute to the central nervous system and cardiac regeneration after ischemic events [55, 56]. Taking into account these observations, Shh pathway might be one of the key pathway linking osteogenesis and angiogenesis during bone repair. We observed that miR-378 simultaneously enhanced neovascularization and osteogenesis in vivo and in vitro. Finally, restoration of Sufu expression levels reversed the effects induced by miR-378, as described above. In addition, this result revealed that miR-378-ASCs-Exos might enhance neovascularization and osteogenesis through the activation of the Shh signaling pathway.

## Conclusion

In summary, our study demonstrated that miR-378-ASCs-Exos exert beneficial effects on GC-induced ONFH by enhancing osteogenesis and angiogenesis. Potential mechanisms involved could be related to miR-378-mediated suppression of Sufu expression and activation of the Shh signaling pathway. Our findings provide a promising therapeutic strategy for GC-induced ONFH.

## Data Availability

The data that support the findings of this study are available from the corresponding author upon reasonable request.

## Abbreviations

Sufu: Suppressor of fused
GC: Glucocorticoid
ONFH: Osteonecrosis of the femoral head
ASCs: Adipose-derived stem cells
BMSCs: Bone marrow stromal cells
HUVECs: Human umbilical vein endothelial cells
ASCs-Exos: ASC-derived exosomes
DEX: Dexamethasone
Shh: Sonic Hedgehog
miRNAs: MicroRNAs
FBS: Fetal bovine serum
α-MEM: Essential Medium Alpha Medium
TEM: Transmission electron microscopy
Ptch: Patched
Smo: Smoothened
RT-qPCR: Quantitative real-time PCR
